# Nicotinic Acetylcholine Receptor α9 and α10 Subunits Are Expressed in the Brain of Mice

**DOI:** 10.3389/fncel.2017.00282

**Published:** 2017-09-12

**Authors:** Olena Lykhmus, Larysa P. Voytenko, Katrin S. Lips, Ivonne Bergen, Gabriela Krasteva-Christ, Douglas E. Vetter, Wolfgang Kummer, Maryna Skok

**Affiliations:** ^1^Laboratory of Cell Receptors Immunology, Palladin Institute of Biochemistry (NAS Ukraine) Kiev, Ukraine; ^2^Laboratory of Experimental Trauma Surgery, Justus-Liebig University Giessen Giessen, Germany; ^3^Institute for Anatomy and Cell Biology, University Homburg/Saar Homburg, Germany; ^4^Department of Neurobiology and Anatomical Sciences, University of Mississippi Medical Center Jackson, MS, United States; ^5^Institute for Anatomy and Cell Biology, Justus-Liebig University Giessen Giessen, Germany; ^6^German Center for Lung Research (DZL) Giessen, Germany

**Keywords:** α7, α9, α10 nicotinic acetylcholine receptors, brain, sandwich ELISA, immunohistochemistry, RT-PCR

## Abstract

The α9 and α10 nicotinic acetylcholine receptor (nAChR) subunits are likely to be the evolutionary precursors to the entire cys-loop superfamily of ligand-gated ion channels, which includes acetylcholine, GABA, glycine and serotonin ionotropic receptors. nAChRs containing α9 and α10 subunits are found in the inner ear, dorsal root ganglia and many non-excitable tissues, but their expression in the central nervous system has not been definitely demonstrated. Here we show the presence of both α9 and α10 nAChR subunits in the mouse brain by RT-PCR and immunochemical approaches with a range of nAChR subunit-selective antibodies, which selectivity was demonstrated in the brain preparations of α7−/−, α9−/− and α10−/− mice. The α9 and α10 RNA transcripts were found in medulla oblongata (MO), cerebellum, midbrain (MB), thalamus and putamen (TP), somatosensory cortex (SC), frontal cortex (FC) and hippocampus. High α9-selective signal in ELISA was observed in the FC, SC, MO, TP and hippocampus and α10-selective signal was the highest in MO and FC. The α9 and α10 proteins were found in the brain mitochondria, while their presence on the plasma membrane has not been definitely confirmed The α7-, α9- and α10-selective antibodies stained mainly neurons and hypertrophied astrocytes, but not microglia. The α9- and α10-positive cells formed ordered structures or zones in cerebellum and superior olive (SO) and were randomly distributed among α7-positive cells in the FC; they were found in CA1, CA3 and CA4, but not in CA2 region of the hippocampus. The α9 and α10 subunits were up-regulated in α7−/− mice and both α7 and α9 subunits were down-regulated in α10−/− mice. We conclude that α9 and α10 nAChR subunits are expressed in distinct neurons of the mouse brain and in the brain mitochondria and are compensatory up-regulated in the absence of α7 subunits.

## Introduction

Nicotinic acetylcholine receptors (nAChRs) are ligand-gated ion channels mediating fast synaptic transmission in muscles and autonomic ganglia (Skok, 2002; Kalamida et al., 2007), regulating transmitter release in the brain (Gotti et al., 2009) and controlling vital cellular functions like survival, proliferation or adhesion in many excitable and non-excitable cells (Kawashima and Fujii, 2008). Structurally, the nAChRs are homo- or heteropentamers composed of alpha (α1-α10) and beta (β1-β4) subunits; muscular nAChRs also contain γ, δ and ɛ subunits (Zouridakis et al., 2009). The α7 and α9 subunits are able to form homomeric receptors with five potential ACh binding sites and are considered to be the most evolutionary ancient of the nAChRs (Ortells and Lunt, 1995). All other subunits are combined in various combinations to form heteromeric nAChRs (α3β2, α3β4, α3α5β4, α4β2, etc.) with the common stoichiometry α_2_β_3_ that produces two ACh binding sites formed at the border of alpha and beta subunits (Zouridakis et al., 2009). The α7 and α9 subunits can also be parts of heteromeric nAChRs. Several studies revealed the presence of α7β2 nAChRs in the brain and autonomic ganglia (Khiroug et al., 2002; Moretti et al., 2014). The α9 subunits are known to combine with α10 subunits to form α9α10 nAChRs with the kinetic properties slightly different from homomeric α9 nAChRs (Plazas et al., 2005). Recent data suggested a functional interaction between α7, α9 and α10 nAChR subunits (Mishra et al., 2010). Two lines of evidence demonstrated up-regulation of α9 nAChR subunits expression upon down-regulation of α7 nAChRs (Grau et al., 2007) or in α7 knockout (KO) mice (Koval et al., 2011) which could be a compensatory event suggesting similar functions of α7- and α9-containing nAChRs in some cells. Indeed, we have found that α9 nAChRs regulated B lymphocyte proliferation similarly to α7 nAChRs (Koval et al., 2011).

The α9(α10) nAChR is one of the most recently discovered nAChR subtypes. Initially, these receptors were found in the hair cells of the inner ear and regulate auditory functions (Elgoyhen et al., 1994). Later, their expression was observed in many other locations and tissues (Peng et al., 2004; Chernyavsky et al., 2007; Hecker et al., 2009; Mikulski et al., 2010; Chikova and Grando, 2011; Koval et al., 2011; St-Pierre et al., 2016) including dorsal root ganglia (Lips et al., 2002). Valuable data on physiological functions of α9(α10) nAChRs have been obtained using α9−/− (Vetter et al., 1999) and α10−/− mice (Vetter et al., 2007) and α9-specific toxins (McIntosh et al., 2005). The α9 nAChRs were shown to be involved in regulation of chronic pain (Vincler and McIntosh, 2007; Romero et al., 2017). Surprisingly, no α9 mRNA was found in the brain by *in situ* hybridization (Elgoyhen et al., 1994), although expression of α9 nAChR subunits was later shown immunohistochemically in the brainstem medulla and hippocampus of piglets and mice (Vivekanandarajah et al., 2015, 2016).

Here we employed RT-PCR and a range of immunochemical approaches to demonstrate that α9 and α10 nAChR subunits are expressed in distinct brain areas of C57Bl/6 mice.

## Materials and Methods

### Animals and Reagents

The brain studies were performed in female C57Bl/6 mice, 2–3 months of age. The wild type (WT) mice were kept in the animal department of the Palladin Institute of Biochemistry, Kiev, while mutant mice lacking the α7 (Orr-Urtreger et al., 1997), α9 (Vetter et al., 1999) or α10 (Vetter et al., 2007) nAChR subunits of either sex were kept in the animal facility of either the Justus-Liebig-University, Giessen or the University of Mississippi Medical Center, Jackson. All animals were housed in a quiet, temperature-controlled room (22–23°C) and were provided with water and dry food pellets *ad libitum*. Mice were sacrificed by cervical dislocation to remove the brain.

The α7−/− mice were generated on the C57Bl/6 background (Orr-Urtreger et al., 1997), while α9−/− mice are the product of 129/SvDNA used to target the α9 locus in W9.5 embryonic stem cells. Following identification of correct homologous recombination (via Southern blot), embryonic stem cells were injected into blastocysts derived from 129/SvEv mice and resultant offspring were then bred to both CBA/CaJ mice and 129/SvEv mice to establish the original α9 null lines (Vetter et al., 1999). Early work did not reveal a background contribution to the biological processes examined (anatomical and functional analyses of the inner ear), and ultimately the α9 null line was maintained only on the CBA/CaJ background. Mice were backcrossed to CBA/CaJ WT mice for a total of N7 generations.

All procedures of this study were performed in accordance with the European Communities Council Directive of 24 November 1986 (86/609/EEC), German guidelines, and NIH/USDA guidelines for the care and use of animals in laboratory research and conformed to the guidelines of the Animal Care and Use Committee of Palladin Institute and were approved by the IACUC Protocol 1/7-421. All efforts were made to minimize the number of animals used.

All reagents were of chemical grade and were purchased from Sigma-Aldrich unless specially indicated. Rabbit antibodies, used for mouse brain studies, against α3(181–192), α4(181–192), α5(180–191), α7(179–190), α7(1–208), α9(11–23) and α10 (404–417) nAChR fragments were obtained and characterized by us previously (Skok et al., 1999; Lips et al., 2002; Koval et al., 2004, 2011; Lykhmus et al., 2010). The antibodies were either biotinylated according to standard procedure (Harlow and Lane, 1988) or conjugated to Atto-488 (Fluka-Sigma Aldrich, Germany) as recommended by the manufacturer. Rabbit antibodies against glial fibrillary acidic protein (GFAP) were from Dako (Agilent Technologies, USA); goat anti-rabbit IgG, Alexa 488-conjugated, was from Invitrogen (Germany); goat antibodies against Iba-1 (PA5–18039) were from Thermo Scientific (France). The α9-specific α-conotoxin PeIA (McIntosh et al., 2005) was synthesized in Shemyakin-Ovchinnikov Institute of Bioorganic Chemistry (Moscow, Russian Federation; Koval et al., 2011) and was a kind gift of Prof. V. Tsetlin. It was biotinylated by a standard procedure (Harlow and Lane, 1988).

### Procedures for Brain Samples Preparation

To prepare the brain detergent lysates, we used either the whole brains or dissected specified regions with reference to mouse brain atlas (Paxinos and Franklin, 2001): frontal cortex (FC), somatosensory cortex (SC), cerebellum, putamen-thalamus, midbrain (MB), hippocampus and medulla oblongata (MO). Whole brains or dissected regions were homogenized with a glass homogenizer, lysed in detergent-containing buffer (0.01 M Tris-HCl, pH 7.4, 1 M NaCl, 1 mM EGTA, 1% Triton X-100) for 45 min on ice and centrifuged at 25,000 *g*.

Mitochondria were isolated from the brain by differential ultracentrifugation according to standard published procedures (Sottocasa et al., 1967; Gergalova et al., 2012) frozen at −20°C and thawed. The pellet obtained after the first centrifugation of the primary brain homogenate (10 min at 1500× *g*) was considered depleted of mitochondria. Mitochondria, the whole brain and the brain depleted of mitochondria preparations were treated with lysing buffer (0.01 M Tris-HCl, pH 8.0; 0.14 NaCl; 0.025% NaN_3_; 1% Tween-20 and protease inhibitors cocktail) for 2 h on ice upon intensive stirring. The resulting lysates were cleared by centrifugation (20 min at 20,000× *g*). The protein concentration in the cleared lysates was established by using the BCA Protein Assay kit (Thermo Scientific, Rockford, IL, USA).

For immunohistochemical experiments, brains were fixed in 4% paraformaldehyde for 48 h and washed in PBS. Forty micrometer thick coronal sections were cut using a vibratome (Leica, Germany) and collected in PBS.

### Sandwich ELISA Assays

The purity of mitochondrial vs. mitochondria-depleted brain fractions was assessed by Sandwich ELISA as described previously (Uspenska et al., 2017). Nighty-six-well plates (Nunc Maxisorb, Roskilde, Denmark) were coated with anti-lamin B1, anti-voltage-dependent anion channel (anti-VDAC) or anti-inositol-requiring enzyme-1α (anti-IRE-1α; 200 μg/ml in 50 μl of PBS; 2 h at 37°C), blocked with 1% BSA/PBS (1 h) and detergent lysates of either mitochondrial or mitochondria-depleted fractions (100 μg/ml) were applied for 2 h at 37°C. Then, the plates were rinsed with water and the bound antigen was revealed with biotinylated anti-lamin B1, anti-VDAC or anti-IRE-1α (50 μg/ml in 50 μl of PBS; overnight at 4°C) followed by NeutrAvidin-peroxidase conjugate and *o*-phenylenediamine-containing substrate solution.

To determine the level of α7, α9 or α10 nAChR subunits within the brain detergent lysates, immunoplates (Nunc MaxiSorp) were coated with rabbit antibody raised against α7(1–208) and capable to recognize a wide spectrum of nAChR subunits due to structural homology in their extracellular domains (20 μg/ml), blocked with 1% BSA and the brain preparations of either WT or KO mice were applied into the wells (1 μg of protein per 0.05 ml per well) for 2 h at 37°C. Plates were washed with water and incubated for additional 2 h with biotinylated α3(181–192)-specific (1:100), α4(181–192)-specific (1:80), α7(179–190)-specific (1:80), α9(11–23)-specific (1:150), α10 (404–417)-specific (1:300), β2(190–200)-specific (1:50) or β4(190–200)-specific antibody (1:100) (assuming the initial antibody concentration was 2 mg/ml) that were visualized using a streptavidin-peroxidase conjugate and an *o*-phenylenediamine-containing substrate solution. The optical density was read at 490 nm by Stat-Fax 2000 ELISA Reader (Awareness Technology, Westport, CT, USA).

### Immunohistochemistry and Confocal Microscopy of Brain Sections

The non-specific binding of antibodies to the brain sections was blocked with 1% BSA in PBS (30 min, room temperature). The following antibody combinations were applied for staining. All procedures were performed at room temperature.

#### Double Staining for the nAChR Subunits and Nuclei

The slides were incubated with biotinylated α7(179–190)-specific (1:100), α9(11–23)-specific (1:50) or α10(404–417)-specific (1:1000) antibodies overnight, washed with PBS and incubated with Extravidin-Cy3 (1:200) and DAPI (1%) in 1% BSA-containing PBS for 1 h.

#### Double Staining for α7 and Either α9 or α10 nAChR Subunits

The slides were incubated with biotinylated α9(11–23)-specific (1:50) or α10(404–417)-specific (1:1000) antibodies overnight, washed with PBS and incubated with Extravidin-Cy3 (1:200) and Atto-488-labeled α7(179–190)-specific antibody (1:100) in 1% BSA-containing PBS for 1 h.

#### Double Staining for α7, α9 or α10 nAChR Subunits and Iba-1

The slides were incubated with biotinylated α7(179–190)-specific (1:100), α9(11–23)-specific (1:50) or α10(404–417)-specific (1:1000) antibodies overnight, washed with PBS and incubated with Extravidin-Cy3 (1:200) and Atto-488-labeled Iba-1-specific antibody (1:100) in 1% BSA-containing PBS for 1 h.

#### Double Staining for α7, α9 or α10 nAChR Subunits and GFAP

The slides were incubated with GFAP-specific antibody (1:500) in 1% BSA-containing PBS overnight followed by Alexa-488-labeled anti-rabbit IgG (1 h). Then the slides were washed with PBS and incubated with biotinylated α7(179–190)-specific (1:100), α9(11–23)-specific (1:50) or α10(404–417)-specific (1:1000) antibodies followed by Extravidin-Cy3 (1:200) for 1 h. This order of stainings was employed to overcome the potential binding of Alexa 488-labeled anti-rabbit IgG with biotinylated rabbit antibodies against nAChR subunits.

#### Staining with α-conotoxin PeIA

The slides were incubated with biotinylated α-conotoxin PeIA (25 nM) overnight, washed with 2 ml PBS (3 × 20 min at RT with shaking) and incubated with Extravidin-Cy3 (1:200) for 1 h followed by similar washing procedure.

All slides were embedded in MOWIOL-DABCO and examined under Zeiss LSM 510 Meta confocal laser scanning microscope. The brain regions were identified according to Paxinos and Franklin (2001).

### RT-PCR

RNA of brain regions of five male 15 weeks old C57BL6 wildtype mice (*n* = 5) were isolated by using the TRIzol^®^ reagent (Invitrogen, Darmstadt, Germany). Therefore, tissue covered with 1 mL TRIzol^®^, homogenized and incubated for 5 min at room temperature. 200 μL of chloroform was added and centrifuged at 14,000 rpm for 15 min at 4°C. The RNA containing layer was collected, added to 500 μL isopropanol and incubated for 15 min at room temperature. After centrifugation (15 min, 14,000 rpm) the RNA pellet was washed with ethanol and finally resuspended with RNase free water. The Quantitect kit (Qiagen, Hilden, Germany) was used for removal of contaminating DNA and subsequent cDNA synthesis according to the manufacturer’s protocol. The cDNAs were amplified with subunit α9 and α10 gene specific primer pairs (α9 with an amplified product length of 122 bp, forward: CAGGTCACGCTCTCCCAG, reverse: CCGTCATACTGGTCTCGATCC, accession number NM_001081104; α10: product length of 140 bp, forward: GGCAGACACAGACCAGACTC, reverse: GGTCCCAATGTAGGTAGGCG, accession number NM_001081424). β-actin was used as reference gene (product length: 165 bp, forward: TGTTACCAACTGGGACGACA, reverse: GGGGTGTTGAAGGTCTCAAA, accession number NM_007393). All primers were intron spanning and synthesized by MWG Biotech, Ebersberg, Germany. Real-time RT-PCR was performed in a Lightcycler (Roche, Grenzach, Germany) using the QuantiFast SYPR Green PCR Kit (Qiagen). Therefore, 5 μL of the Mastermix, 1 μL cDNA, 3.8 μL water and 0.2 μL of forward and reverse primer were added and incubated 5 min at 95°C, then 40 cycles with 10 s at 95°C, 30 s at 60°C were conducted. The PCR products were separated by electrophoresis on a 1.2% TRIS-acetate-EDTA gel. Control reactions omitted DNA template or reverse transcriptase.

### Statistical Analysis

We used five mice per genotype in Sandwich ELISA with the whole brain preparations and the brains of four WT mice for analyzing separate brain regions. Each ELISA assay has been performed in triplicates. The mean values for individual mice were used for statistical analysis using Student’s *t*-test. The data are presented as M ± SE; *p < 0.05; ***p < 0.0005.

## Results

To study the presence of α7, α9 and α10 nAChR subunits in the detergent lysates of the mouse brain we at first used the Sandwich ELISA approach, which had been developed in our laboratory and previously employed to reveal other nAChR subtypes in the mouse brain and mitochondria (Lykhmus et al., 2011, 2014). This assay includes coating antibody raised against the whole extracellular domain (1–208) of α7 subunit, able to capture a wide range of nAChR subunits due to substantial structural homology of their extracellular domains, and a detecting biotinylated antibody against a specific epitope of certain subunit. The use of brain samples obtained from α7−/−, α9−/− or α10−/− (KO) mice allowed us to evaluate the subunit selectivity of the assay and to justify its usage in subsequent experiments.

**Figure 1:**
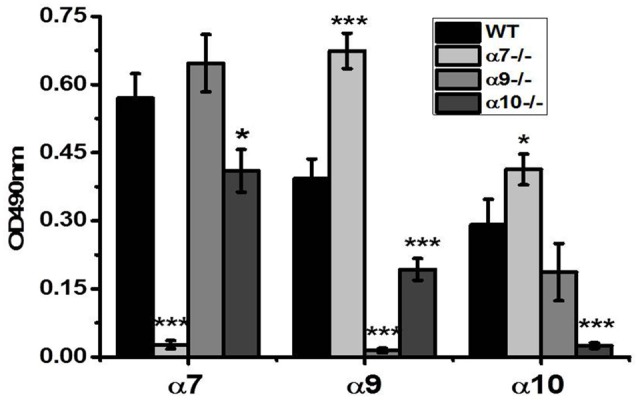
Sandwich ELISA assays with the brain preparations of the wild type (WT), α7−/−, α9−/− and α10−/− mice demonstrating subunit selectivity of α7(179–190)-, α9(11–23)- and α10(404–417)-specific antibodies. The brain samples were captured with α7(1–208)-specific antibody and revealed with α7(179–190)-, α9(190–200)- or α10(404–417)-specific antibodies. The columns correspond to M ± SE; *p < 0.05, ***p < 0.0005 compared to corresponding OD values of WT mice, *n* = 5.

As shown in Figure 1, α7 (179–190)-specific antibody produced a strong signal in the brain samples of the WT mice, a slightly stronger signal in the samples of α9−/− mice, a weaker signal in α10−/− mice and a negligible signal in the samples of α7−/− mice. The α9(11–23)-specific antibody produced an evident signal in the WT, significantly stronger signal in α7−/− samples, a weaker signal in the samples of α10−/− mice and a negligible signal in α9−/− mice. The α10(404–417)-specific antibody produced a negligible signal in α10−/− mice, while α7−/− mice produced higher signal and α9−/− mice lower signal than WT mice. This data clearly indicated that subunit-specific antibodies distinguished between α7, α9 and α10 nAChR subunits and, therefore, the assay could be used to characterize the presence of corresponding subunits in the brain preparations.

According to the data of Figure 1, the brain samples of WT mice contained α7, α9 and α10 subunits. α9 subunits were up-regulated in α7−/− mice and, vice versa, α7 subunits were non-significantly increased in α9−/− mice. The α9 subunits were decreased in α10−/− mice and α10 subunits were non-significantly decreased in α9−/− mice but increased in α7−/− mice.

Previously the presence of α9 nAChR subunits was reported in mitochondria purified from the skin (Chernyavsky et al., 2015) and we found them in liver mitochondria (Uspenska et al., 2017). To find out if α9 or α10 nAChR subunits are present in the brain mitochondria, we fractionated the brain homogenate of the WT and α9−/− mice into mitochondria and mitochondria-depleted fractions.

**Figure 2:**
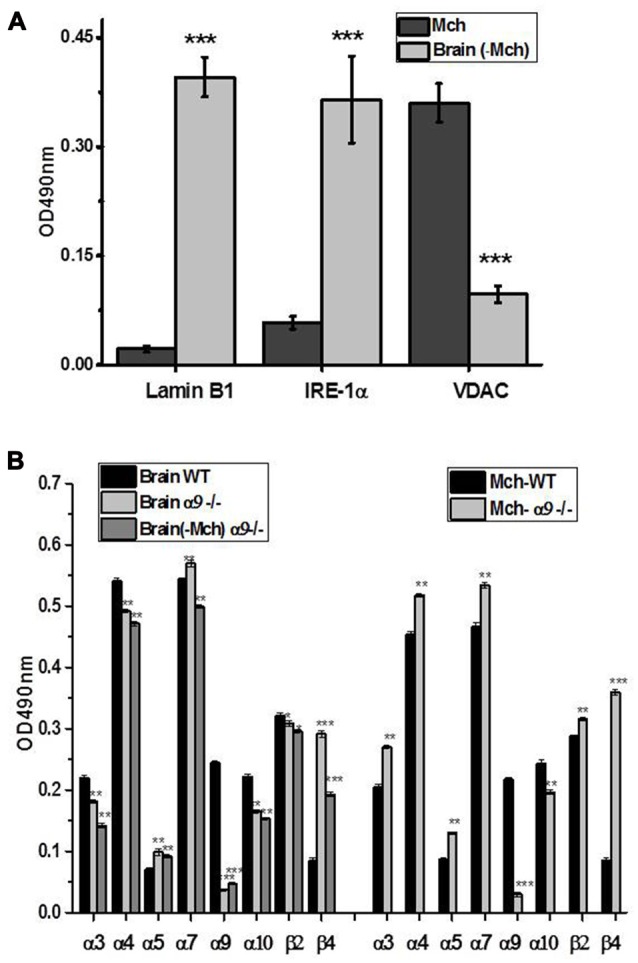
Characterization of mitochondria (Mch) and mitochondria-depleted brain fraction (Brain (-Mch)) **(A)** and subunit composition of nicotinic acetylcholine receptors (nAChRs) in the whole brain, mitochondria or mitochondria-depleted brain fraction of WT and α9−/− mice **(B)**. **(A)** The samples were studied by Sandwich ELISA using the antibodies against voltage-dependent anion channel (VDAC), lamin B1 or inositol-requiring enzyme-1α (IRE-1α). **(B)** The samples were captured with α7(1–208)-specific antibody and revealed with α3(181–192)-, α4(181–192), α5(180–191), α7(179–190)-, α9(190–200)-, α10(404–417)-, β2(190–200) or β4(190–200)-specific antibodies. The columns correspond to M ± SE; **p < 0.005, ***p < 0.0005 compared to corresponding OD values of WT mice, *n* = 5.

The purity and contaminants of mitochondrial and mitochondria-depleted brain fractions was assessed by Sandwich ELISA using the antibodies against mitochondrial marker VDAC (Colombini, 2004), nuclear marker lamin B1 (Gruenbaum et al., 2000) and marker of endoplasmic reticulum IRE-1α (Chen and Brandizzi, 2013). As shown in Figure 2A, the mitochondrial fraction did not contain nuclear marker and contained only trace amounts of IRE-1α, which were abundant in the non-mitochondrial fraction. Correspondingly, only trace amounts of mitochondrial marker VDAC were found in mitochondria-depleted fraction. We did not apply antibodies against plasma membrane markers assuming that plasma membrane nAChRs comprise only a small fraction (about 15%) of the whole cellular pool (Sallette et al., 2005), therefore, potential contamination of mitochondrial fraction with the plasma membranes should play a negligible role.

As shown in Figure 2B, the signal for α9 subunit was found in both brain fractions of the WT but not α9−/− mice. The absence of α9 subunits resulted in the decrease of α10 subunits and significant up-regulation of β4 subunits in both fractions compared to the WT preparations. Interestingly, the α3, α4, α7 and to a lesser degree, β2 subunits were increased in mitochondria of the α9−/− mice but decreased in the rest of the brain compared to the WT, demonstrating the re-distribution of α3-, α4- and α7-containing nAChRs in favor of mitochondria. Therefore, the absence of α9 subunits was compensated by other nAChR subtypes in mitochondria but not in other cellular components of the brain.

**Figure 3:**
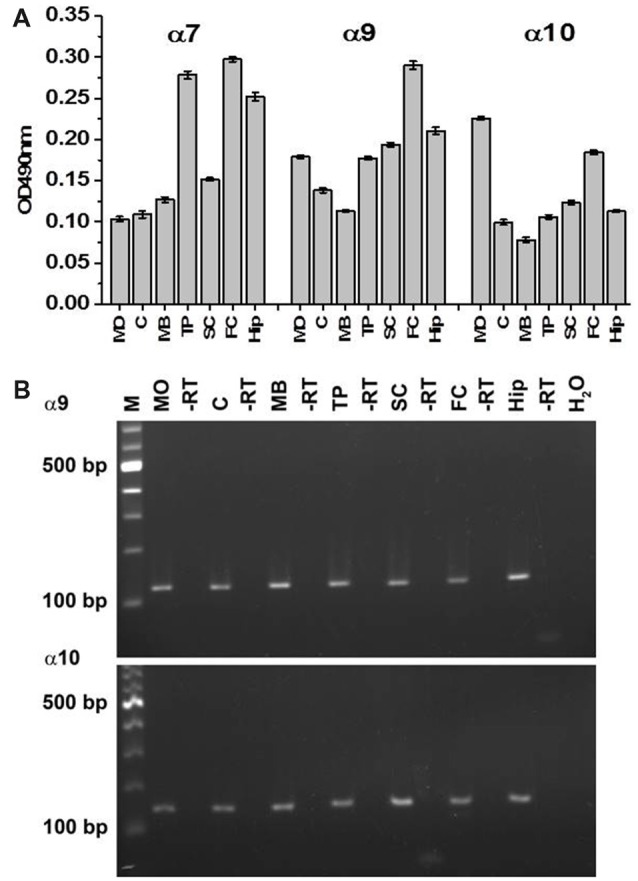
Sandwich ELISA **(A)** and RT-PCR **(B)** of various brain regions of the WT mice. In **(A)**, the brain samples were captured with α7(1–208)-specific antibody and revealed with α7(179–190)-, α9(190–200)- or α10(404–417)-specific antibodies. Each column corresponds to M ± SE of data obtained from five mice per brain region. MO, medulla oblongata; C, cerebellum; MB, midbrain; TP, thalamus and putamen; SC, somatosensory cortex; FC, frontal cortex; Hip, hippocampus; -RT negative control where no enzyme was used during cDNA synthesis, H_2_O, negative control where water was used instead of cDNA; M, marker; bp, base pair.

Similar Sandwich ELISA was performed in detergent lysates of the functionally different regions dissected from the brains of the WT mice. The highest level of α7 subunit was found in the FC, thalamus-putamen (TP) and hippocampus and the lowest level in MO, MB midbrain and cerebellum (Figure 3A). High expression of α9 subunits was observed in the FC, less in the SC, MO, TP and hippocampus and the lowest signal was found in the MB and cerebellum. The α10 subunit was highest in MO and FC and lowest in the MB. These data indicated that α7, α9 and α10 subunits are distributed within the brain in non-uniform and non-similar way.

The data of the antibody staining were further confirmed by RT-PCR performed with the mRNA purified from various brain regions of the WT mice and subunit α9 and α10 gene-specific primer pairs. The amplified products were sequenced by MWG Biotech and identified as subunit α9 and α10 with conformity of 100% compared to the published sequence (α9: NM_001081104, α10: NM_001081424). As shown in Figure 3B, RNA of α9 and 10 nAChR subunits were found in all investigated brain regions supporting the data obtained by Sandwich ELISA.

The whole brain preparations obviously contain proteins and RNA originating from different types of cells: neurons, neuroglia, vascular endothelium and remaining blood cells. To identify the location of α7, α9 and α10-containing nAChRs in the brain, we performed immunohistochemical staining and confocal microscopy studies of mouse brain sections applying fluorescently labeled nAChR subunit-specific antibodies in various combinations and in combination with the antibodies against cellular markers for microglia or astrocytes.

**Figure 4:**
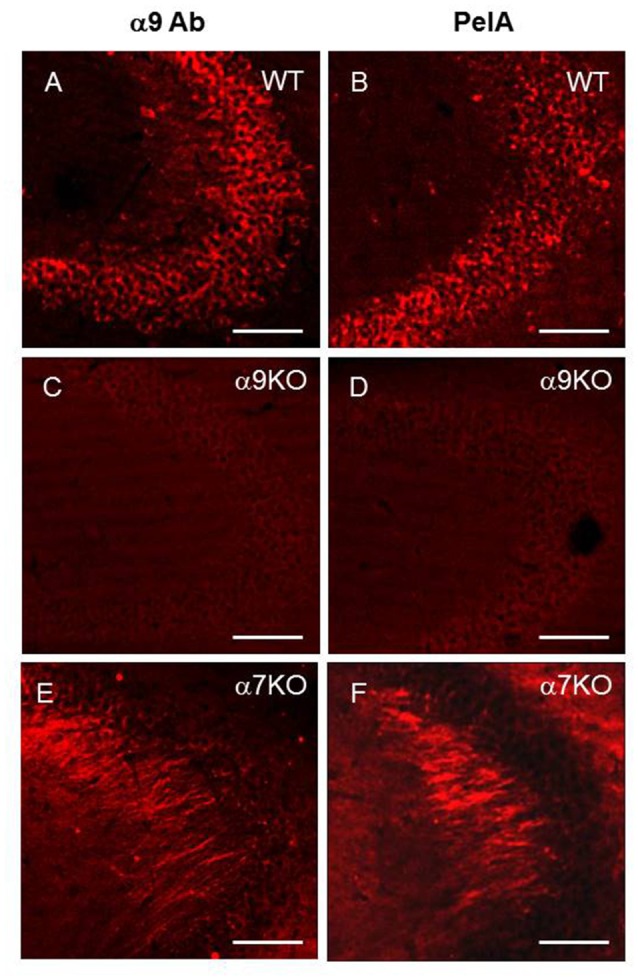
α9-specific staining in the hippocampal CA3 region of the WT **(A,B)**, α9−/− **(C,D)** and α7−/− **(E,F)** mice with either α9-selective antibody **(A,C,E)** or α-conotoxin PeIA **(B,D,F)**, bar is 100 μm.

To prove the α9(11–23)-specific antibody selectivity in immunohistochemical studies, we compared the staining patterns of the hippocampus of the WT and α9−/− mice obtained with either the α9(11–23)-specific antibody or α-conotoxin PeIA. Conotoxin PeIA was shown to display a 260-fold higher selectivity for α9α10 nAChRs compared with α7 receptors with IC50 in nanomolar range for recombinant α9α10 and WT hair cell nAChRs, respectively (McIntosh et al., 2005). As shown in Figure 4, the antibody and conotoxin PeIA produced very similar staining in the CA3 region of the hippocampus, which was not found in the brain of α9−/− mice and was significantly increased in α7−/− mice.

**Figure 5:**
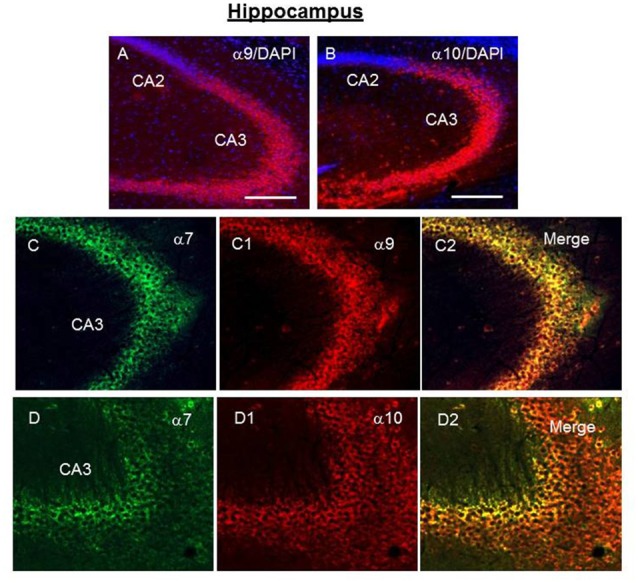
Immunochemical staining of the CA2-CA3 regions of hippocampus of the WT mouse. Green—α7-specific, red—α9- or α10-specific staining, blue—DAPI (cell nuclei) with α9- or α10-selective antibody and DAPI **(A,B)** and with α7- and α9-selective **(C,C1,C2)** or α7- and α10-selective antibodies **(D,D1,D2)**. **(A,B)** Bar is 200 μm; **(C,D)** bar is 100 μm.

**Figure 6:**
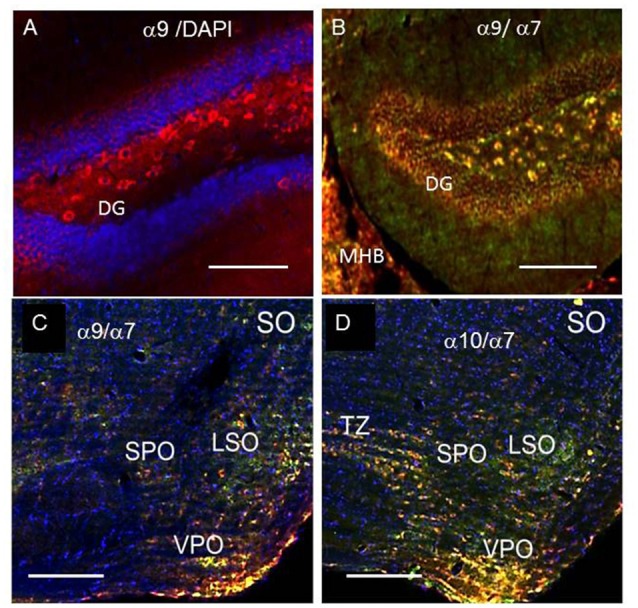
α7- (green) and α9- or α10-specific (red) staining in the dentate gyrus (DG, **A,B**) and superior olive (SO, **C,D**) of the WT mice, blue—DAPI (cell nuclei). MHB, medial habenula; LSO, lateral superior olive; SPO, superior; VPO, ventral periolivary nuclei; TZ, corpus trapezoid. In **(A,B)** bar is 100 μm, in **(C,D)** 200 μm.

Staining for α9 and α10 nAChR subunits was observed in CA1, CA3 and CA4, but not in CA2 region of the hippocampus (Figures 5A,B). In CA3, α9-selective antibody staining was found predominantly in the *strata pyramidale, lucidum* and *radiatum* (Figures 5C,C1,C2) while α10-selective staining was located in the *strata pyramidale* and *oriens* (Figures 5D,D1,D2). The α7-selective (green) staining was found in the nerve fibers coming from *stratum radiatum* (Figure 5D). In the hippocampus of α7−/− mice, these fibers became strongly α9-positive (Figures 4E,F) that was in accord with the increased α9-selective signal found in ELISA (Figure 1). In the brain sections of WT mice, these nerve fibers demonstrated the overlap of α7- (green) and α9-selective (red) staining (Figure 5D). Therefore, the α9 subunits seemed to be present in the fibers and to be significantly up-regulated in the absence of α7 subunits. The red and green staining often overlapped in the pyramidal layer as well suggesting the presence of closely associated α7, α9 and α10 nAChR subunits. The α9- and α10-positive cells were also found in the dentate gyrus (DG; Figures 6A,B) and in SO, where they were concentrated in certain zones including the trapezoid body (the ventral acoustic stria; Figures 6C,D).

**Figure 7:**
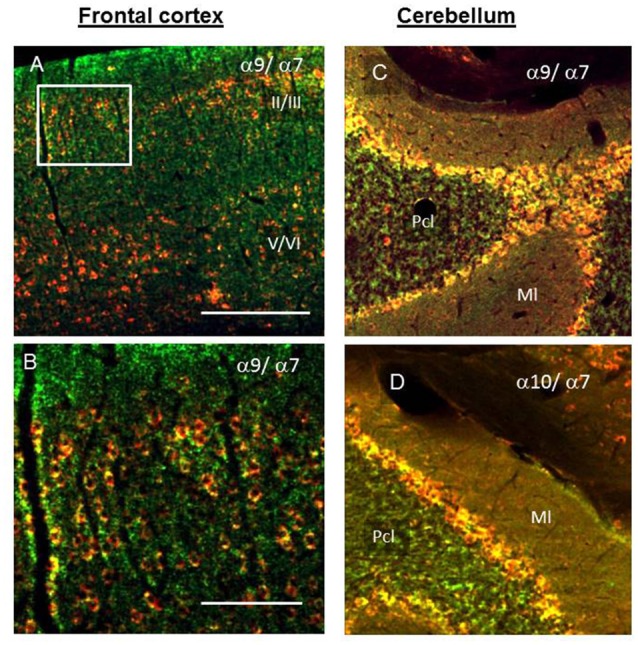
The presence of α7 (green), α9 and α10 (red) nAChR subunits in the II/III and V/VI layers of SC (**A,B**, insert); cortical layers specification according to Ahissar and Staiger, 2010) and cerebellum **(C,D)**. Pcl, Purkinje and granule cell layers; Ml, molecular layer. Yellow—merge of the red and green staining. In **(A,C,D)** bar is 200 μm, in **(B)** 100 μm.

In the FC, α9- and α10-positive cells were found within II-VI cortical layers where their staining overlapped with the α7-selective staining in the external and internal pyramidal cells layers (Figures 7A,B). In the cerebellum, the α9/α10- and α7-selective signals were co-localized in the Purkinje and granular layers and much less in the molecular layer (Figures 7C,D). The α9- and α10-positive cells were also found in the putamen and MO, where they were randomly distributed among α7-positive cells (data not shown).

**Figure 8:**
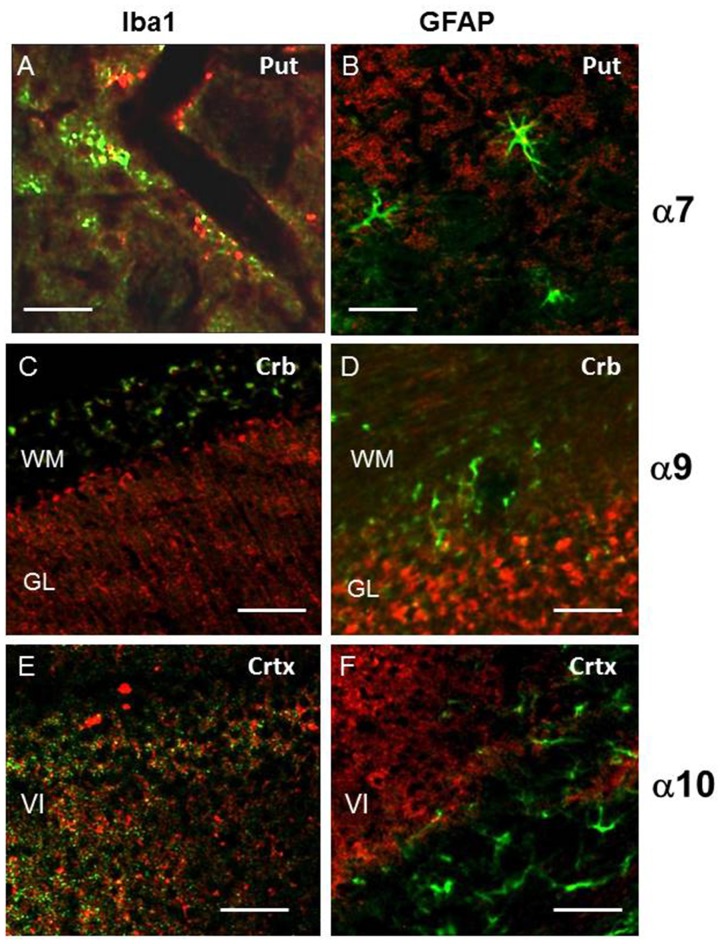
The double staining for α7, α9 or α10 nAChR subunits (red) and markers of microglia (Iba1, green, **A,C,E**) or astrocytes glial fibrillary acidic protein (GFAP, green, **B,D,F**) in the cerebellum (Crb), cortex (Crtx) or putramen (Put). Abbreviations: WM, white matter; GL, granular layer of cerebellum; V, internal pyramidal layer; VI, multiform layer of cortex. In **(A)** bar is 20 μm, in **(B–F)** 50 μm.

Next, brain sections were double-stained with α7-, α9- or α10-selective antibodies and the antibodies against either Iba1 to label the microglia cells or GFAP, a specific marker of activated astrocytes. As shown in Figures 8C–F, Iba1-specific and nAChR-specific antibodies, as well as GFAP-specific and nAChR-specific antibodies stained different cells in the cortex and cerebellum; no overlap between red and green labels was found. In contrast, some activated hypertrophic astrocytes with well-developed processes that were found mostly in putamen, in the cortical multiform layer (layer VI, by Ahissar and Staiger, 2010) and in the medulla were co-stained with GFAP-specific and nAChR-specific antibodies and some microglia cells within putamen were co-stained with Iba1-specific and α7-selective antibodies (Figures 8A,B). In all cases, GFAP-positive astrocytes were surrounded with α7-, α9- or α10-positive cells. This data indicated that α7, α9 and α10 nAChR subunits present in the brain are mostly not located in astrocytes or microglia. However, hypertrophic GFAP-positive astrocytes do express α7-, α9- and α10-containing nAChRs and some microglia cells do express α7 nAChRs.

## Discussion

Initial studies demonstrated the α9 nAChR subunit expression in rat cochlea hair cells (Elgoyhen et al., 1994). Further, this type of receptor was found in lymphocytes (Peng et al., 2004; Koval et al., 2011), adrenal medullary tissue (Colomer et al., 2010), breast epithelial cells (Lee et al., 2010), alveolar macrophages (Mikulski et al., 2010), tracheal epithelium (Hollenhorst et al., 2012), bronchial cells (Chikova and Grando, 2011), keratinocytes and their mitochondria (Chernyavsky et al., 2007, 2015), chondrocytes, adipocytes and osteoblasts (Zablotni et al., 2015), monocytes and neutrophils (Jiang et al., 2016). The involvement of α9 nAChRs in regulating chronic pain (Vincler and McIntosh, 2007; McIntosh et al., 2009; Hone et al., 2017) and their expression in dorsal root ganglia (Lips et al., 2002) demonstrated their presence in nerve cells. However, based on the initial studies (although performed in a different strain of mice than that used here (Elgoyhen et al., 1994)), α9 nAChRs were considered to be absent in the brain. Those experiments (where one of us, DEV, was a co-author) were performed in the brain cryostat sectioned material using ^35^S, ^33^P “double labeled” riboprobes, followed by exposure to X-ray film. A high stringency wash (above 80°C) has been applied to ensure visualizing only the most robust hybridization events. No α9 expression was seen with these probes, which showed a robust label in the inner ear; therefore, this line of work was not further pursued. The same was true when α10 subunit has been cloned and characterized. In the present manuscript, we used immunochemical approaches with the α9- and α10-selective antibodies and RT-PCR with subunit α9 and α10 gene-specific primer pairs. Taken together, the data obtained provide a convincing proof for the α9 and α10 RNA and protein expression in the brain of mice. Recently, expression of α9 nAChR subunits was found immunohistochemically in the brainstem medulla and hippocampus of piglets and mice (Vivekanandarajah et al., 2015, 2016) that supports our data.

We show the presence of α9- and α10-selective immunostaining in both brain detergent lysates and brain sections. Taking into account the substantial homology of nAChR alpha subunits and potential antibody cross-reactivity (Moser et al., 2007), the antibodies used in our studies were tested with the brain preparations of α7−/−, α9−/− or α10−/− mice to demonstrate their selectivity among α7, α9 and α10 nAChR subunits in ELISA. The α9-selective antibody staining in immunohistochemistry was similar to that of conotoxin PeIA and was absent in the brains of α9−/− mice. In addition, confocal microscopy images showed clear difference in the staining patterns of α7(179–190)-, α9(11–23)- and α10(404–417)-specific antibodies supporting their subunit selectivity.

In contrast to the α7-containing nAChRs, which are widely distributed throughout the brain, possibly due to the universal functions of this nAChR subtype in regulation of cell survival and proliferation (Resende and Adhikari, 2009; Lykhmus et al., 2014), the α9- and α10-positive cells are much more rare and form ordered structures or zones, which possibly reflects their specific functions in different brain regions. For example, the patterns of α9- and α10-labeling found in the cerebellum morphologically resemble the Purkinje cells—a class of GABAergic neurons, which send inhibitory projections to the deep cerebellar nuclei and constitute the sole output of all motor coordination in the cerebellar cortex (Ito, 2002). Therefore, α9- and α10-containing nAChRs may be involved in regulating motor coordination. No behavioral data have yet shown this, although it was found that MLA-sensitive non-α7 nAChRs are involved in regulating motor coordination in α7−/− mice (Welch et al., 2013) that may be an indirect evidence to prove our suggestion. The presence of α9α10 nAChRs in GABAergic neurons is of particular interest, because association between the nicotinic cholinergic and GABAergic systems in the cochlea has been suggested (Turcan et al., 2010). In addition, two selective antagonists of α9α10 nAChRs, conotoxins RgIA and Vc1.1, were shown to be also potent GABA-B agonists but did not bind to cloned GABA-B receptors expressed in HEK cells or *Xenopus* oocytes (Hone et al., 2017) that may suggest the functional interaction of α9α10 nAChR and GABA receptors.

Another interesting location of α9 and α10 nAChR subunits in the brain is the SO, specifically the ventral periolivary region. This area contains the olivocochlear efferent neurons projecting to cochlear hair cells, which themselves are well known to express α9α10 nAChRs (Elgoyhen and Katz, 2012). The loss of α9 or α10 nAChR subunits in α9−/− and α10−/− mice resulted in significant changes of efferent fiber presynaptic terminal morphology and innervation patterns under outer hair cells, as well as a change in efferent innervation density to the inner hair cell region (Vetter et al., 1999, 2007). Additionally, vesicle recycling/trafficking machinery changes occurred in α9−/− mice that suggested a bidirectional information flow between the target of the neural innervation (the hair cells) and the presynaptic terminal (Murthy et al., 2009). According to our data, the α9 and α10 nAChR subunits are found in the trapezoid body and ventral periolivary nuclei involved in analyzing the auditory information (Waxman, 2013). Therefore, it cannot be excluded that α9α10-positive hair cells receive innervation from α9α10-positive cells in the SO and the nAChR subunit composition of the target hair cells corresponds to that expressed by the source of their innervation, underlying the way of their development in ontogenesis. Interestingly, while innervation to the cochlear hair cells was abnormal, no discernible defects in collateral fiber innervation to the cochlear nucleus (a target of collaterals from the olivocochlear cells of the SO) was found in the α9−/− mice (Brown and Vetter, 2009).

We observed an increase of α9-selective staining in the hippocampus of α7−/− mice by both ELISA and immunohistochemistry. This suggests that α9 nAChRs can compensate for the absence of α7 nAChRs in the brain, similar to what was previously shown for the rat lung (Grau et al., 2007) or mouse B lymphocytes (Koval et al., 2011). The absence of α9 nAChR subunits resulted in up-regulation of α3-, α4- and α7-containing nAChR subtypes in the brain mitochondria, but not in the rest of the brain, demonstrating the importance of α9 nAChRs for these intracellular organelles. Previously we reported that mitochondrial nAChRs are involved in regulating the inner (mitochondria-driven) pathway of apoptosis (Gergalova et al., 2012, 2014) and that multiple nAChR subtypes expressed in mitochondria ensure the protection from apoptogenic factors of different nature (Lykhmus et al., 2014). The presence of α9 nAChRs in mitochondria found in distinct brain cells may respond to special functional or metabolic requirements of these cells. It is also known that the main part of nAChRs produced by the cell constitute an intracellular pool and only a small portion is expressed on the cell surface (Sallette et al., 2005). It could not be excluded, therefore, that α9-containing nAChRs expressed in the brain do not appear on the plasma membrane but are targeted to mitochondria. This would explain the failure to detect functional α9 nAChRs in the brain, while the low expression levels required for mitochondrial function could explain the lack of *in situ* hybridization signal in early experiments examining the expression localization of α9 (Vetter, unpublished data), as well as recently published transcriptome data of Chrna 9 in mice^1^. In the studies described here we did not explore the presence of α9 nAChRs in the plasma membrane of the mouse brain cells. However, we have a preliminary data suggesting that α9 subunits are present in the plasma membrane preparation of the rat brain. This question needs further examination.

In fact, the transcriptome data^1^ showed a significant α9 expression only in the thymus and no expression was found in the spleen. This looks doubtful because both T and B lymphocytes were reported to express α9 (Peng et al., 2004) and, in general, immune cells are considered to be one of the richest sources of this nAChR subtype (Hao et al., 2011). Also, it is quite clear that transcript levels often have little to do with protein levels. It is therefore possible that just because mRNA seems to be almost at background levels, protein expression levels will, depending on localization, turnover, etc., be quite different. No one-to-one correspondence should be assumed between transcript levels and protein levels. In addition, we show here the obvious presence of α9 transcripts in all brain regions studied.

Another important observation is an unexpected interrelation of α7 and α10 nAChR subunits in the brain. The α7 subunits were down-regulated in α10−/− mice (Figure 1) and α10 subunits were often co-localized with α7, but not α9 subunits in immunohistochemistry (e.g., in hippocampus, Figure 5D). The involvement of α9 and α10 subunits in different nAChR subtypes has been already postulated when it was found that α9−/− and α10−/− mice had non-identical phenotypes (Vetter et al., 2007). Further, functional interaction between α7-, α9- and α10-containing nAChRs was suggested to explain the response of the rat mast/basophil cell line RBL-2H3 to nanomolar concentrations of nicotine (Mishra et al., 2010). Our data support these hypotheses and suggest that α10 nAChR subunits can combine with α7 subunits to form “hybrid” α7α10 receptors. The α7 and α9 nAChR subunits belong to the most ancient members of the superfamily and are highly homologous (Ortells and Lunt, 1995). It is quite possible that the α10 subunit can combine with either of them. Further experiments are required to reveal the functional and pharmacological properties of hypothetical α7α10 nAChRs.

## Author Contributions

MS, DEV and WK: substantial contributions to the conception or design of the work. OL, LPV, GK-C, KSL and IB: acquisition, analysis and interpretation of data for the work. MS, OL, LPV, KSL and DEV: drafting the work; MS, DEV and WK: revising it critically for important intellectual content. OL, LPV, GK-C, KSL, IB, DEV, WK and MS: final approval of the version to be published and agreement to be accountable for all aspects of the work in ensuring that questions related to the accuracy or integrity of any part of the work are appropriately investigated and resolved.

## Conflict of Interest Statement

The authors declare that the research was conducted in the absence of any commercial or financial relationships that could be construed as a potential conflict of interest.

## Footnotes

^1^https://www.ncbi.nlm.nih.gov/gene/231252

## Abbreviations

GFAP: glial fibrillary acidic protein
IRE-1α: inositol-requiring enzyme-1α
KO: knockout
nAChR: nicotinic acetylcholine receptor
VDAC: voltage-dependent anion channel
WT: wild type.
